# Laser-Guided Cervical Selective Nerve Root Block with the Dyna-CT: Initial Experience of Three-Dimensional Puncture Planning with an *Ex-Vivo* Model

**DOI:** 10.1371/journal.pone.0069311

**Published:** 2013-07-19

**Authors:** Miriam I. E. Freundt, Manuel Ritter, Mansour Al-Zghloul, Christoph Groden, Hans U. Kerl

**Affiliations:** 1 University of Heidelberg, Medical Faculty Mannheim, Department of Neuroradiology, Theodor-Kutzer-Ufer 1–3, Mannheim, Germany; 2 University of Heidelberg, Medical Faculty Mannheim, Department of Urology, Theodor-Kutzer-Ufer 1–3, Mannheim, Germany; University of Medicine & Dentistry of NJ - New Jersey Medical School, United States of America

## Abstract

**Background:**

Cervical selective nerve root block (CSNRB) is a well-established, minimally invasive procedure to treat radicular cervical pain. However, the procedure is technically challenging and might lead to major complications. The objective of this study was to evaluate the feasibility of a three-dimensional puncture planning and two-dimensional laser-guidance system for CSNRB in an *ex-vivo* model.

**Methods:**

Dyna-CT of the cervical spine of an *ex-vivo* lamb model was performed with the Artis Zee® Ceiling (Siemens Medical Solutions, Erlangen, Germany) to acquire multiplanar reconstruction images. 15 cervical nerve root punctures were planned and conducted with the syngo iGuide® laser-guidance system. Needle tip location and contrast dye distribution were analyzed by two independent investigators. Procedural, planning, and fluoroscopic time, tract length, and dose area product (DAP) were acquired for each puncture.

**Results:**

All 15 punctures were rated as successful with 12 punctures on the first attempt. Total procedural time was approximately 5 minutes. Mean planning time for the puncture was 2.03 (±0.39) min. Mean puncture time was 2.16 (±0.32) min, while mean fluoroscopy time was 0.17 (±0.06) min. Mean tract length was 2.68 (±0.23) cm. Mean total DAP was 397.45 (±15.63) µGy m^2^.

**Conclusion:**

CSNRB performed with Dyna-CT and the tested laser guidance system is feasible. 3D pre-puncture planning is easy and fast and the laser-guiding system ensures very accurate and intuitive puncture control.

## Introduction

Cervical radicular pain is a disabling condition, which affects approximately 0.1% of the population per year [1], [2]. The classic clinical picture includes neck pain with irradiation to the arm and fingers corresponding to the dermatomes involved, paraesthesias in arm and hand in conjunction with diminished muscle tendon reflexes, sensory disturbances and/or motor weakness with dermatomal/myotomal distribution [3], [4]. Cervical radiculopathy is in 20–25% of the cases caused by cervical disk herniation [2]. Nevertheless the most common cause of root compression at the cervical level is narrowing of the foraminal space secondary to spondylarthrosis and the most common level of root compression is C7, followed by C6 [4].

The majority of the patients complaining about cervical radiculopathy due to degenerative etiologies report spontaneous recovery of the symptoms by time, physical therapy and/or oral medication [2], [5], [6], [7]. In a minor part of the patients the natural resolution of the symptoms is not seen. This group, with persistent disabling pain, consequently requires some form of intervention. Cervical spinal surgery is, because of the well-known drawbacks, including perioperative complications, limitation of range of motion, and/or adjacent segment degeneration, reserved for patients in whom non-operative treatment has failed [8], [9].

Over the last decade selective injection of steroid and/or local anesthetic in the nerve root foramen and into the peri-radicular space - known as cervical selective nerve root block (CSNRB) - has become a standard procedure to conservatively treat unremitting cervical radicular pain [10], [11], [12]. The aim of these procedures is to infiltrate near the existing nerve root and/or epidural space at the affected level to reduce inflammation believed to cause the patient’s discomfort. Prior studies demonstrated favorable results in pain relief after a series of selective nerve root blocks [13], [14]. Besides ameliorating radiculopathy due to disk herniation and cervical spinal stenosis [15] CSNRB can bridge to surgery or improve persistent pain after discectomy [16]. Furthermore, the technique can be used both diagnostically and therapeutically [17], [18].

Anesthesiologists have performed the procedure for many years using anatomical landmarks [19] but with an improve in imaging guidance, it is now increasingly within the spectrum of interventional radiologists and neuroradiologists. Besides ultrasound-guidance [15], nowadays these blocks are often performed under fluoroscopy or with CT-guided techniques [15], [20], [21], [22]. Despite attractive features of CT-guided techniques compared to fluoroscopy and ultrasound-guidance, including direct visualization of soft-tissue planes, vital organs, neural and vascular structures and the precise anatomic localization with millimeter accuracy, all previously introduced approaches lack the possibility of detailed 3D puncture planning and 2D laser-guided needle control. In contrast, with the syngo iGuide® laser guidance system for Dyna-CT (Siemens Medical Solutions, Erlangen, Germany) puncture planning can be obtained in a completely rotatable 3D reconstruction of the target area. Needle control is provided through 2D laser-guidance and guided fluoroscopy. Dyna-CT offers new imaging and needle guiding techniques, which have been introduced in different areas of clinical practice for several divers procedures such as adrenal venous sampling [23], endovascular embolization [24], and endovascular interventions [25]. A valuable new addition is the syngo iGuide® laser guiding system (Siemens Medical Solutions, Erlangen, Germany). This software has shown in a prior study that accurate puncture planning can be achieved in percutaneous kidney puncture [26]. Nevertheless, Dyna-CT with syngo iGuide® has never been used for CSNRB. Therefore the aim of this study was, to evaluate the feasibility of CSNRB performed with the Dyna-CT and iGuide® in an *ex-vivo* lamb model.

## Materials and Methods

### Ex- vivo Lamb Model

This cadaveric study was performed according to our institution guidelines for cadaver work. To resemble the human cervical anatomy in our *ex-vivo* model, we used a cervical spine of a lamb with peri-vertebral tissue including autochthonous back muscles (1.6 kg) from a local slaughterhouse (Yalya, Mannheim, Germany). A permission form this slaughterhouse to use these animal parts was obtained.

### Imaging System

All procedures were performed with the Dyna-CT (Siemens Medical Solutions, Erlangen, Germany), a ceiling mounted flat-panel detector-based 3D imaging system. This system consists of an Artis Zee® Ceiling and a free-floating full-carbon interventional table. The 40 × 30 cm flat panel detector with the C-arm is completely free moveable around the interventional table. In addition to projectional X-ray imaging the system is able to acquire large volume CT-like images overtopping standard C-arm based 3D volume imaging.

We used a dedicated program for syngo iGuide® puncture planning to optimize the visualization of the spine with a field of view of 48 cm. The rotation time for Dyna CT was 8 s acquiring 60 frames per second. Source power used was 90 kVP. The images were transmitted in less than one minute to a local workstation (Leonardo, Siemens Medical Solutions, Erlangen, Germany) equipped with the software MMWP VE 40A 3D for 3D rendering and post-image processing. From the acquired data set, slice image reconstructions and a 3D model with a matrix of 512 × 512 × 512 was generated in 48 s, which was displayed in 2D (Multiplanar Reformat, MPR) with a voxel matrix is 512×512 and a slice thickness of 3 mm.

### Puncture Planning with Syngo iGuide®

Syngo iGuide® is a software offering detailed 3D-pre-puncture planning and 2D-laser guidance of the needle during puncture. Thereby, syngo iGuide® clearly displays the length and angulation of the needle path and automatically positions the C-arm for planning the needle trajectory. The integrated laser crosshair projected onto the skin enhances needle guidance by indicating the entry point as well as the angle of the needle. With the possibility to overlay on to live fluoroscopy needle progression can be controlled in one fluid process.

**Figure 1:**
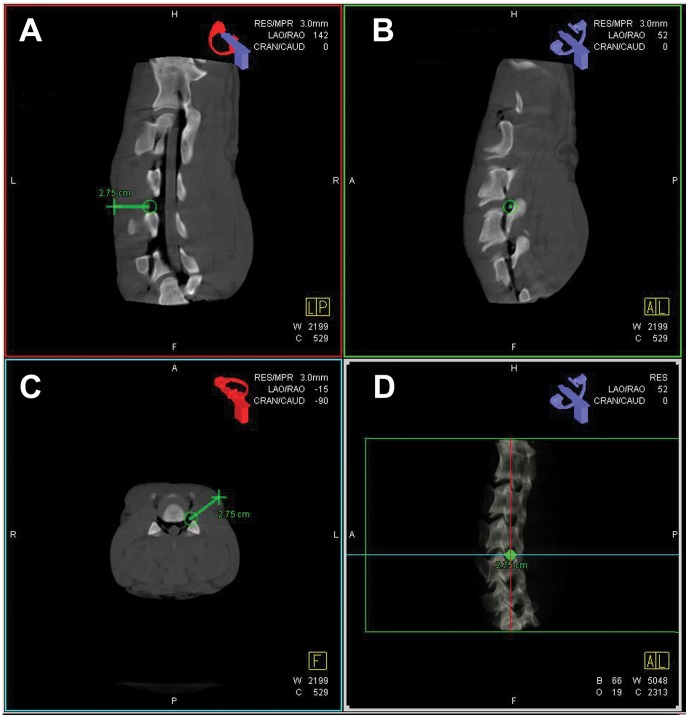
Three-dimensional puncture planning using syngo iGuide®. Three-dimensional puncture planning using syngo iGuide® with indication of the planned needle trajectory in two angulated lateral (**A** and **B**) views and the axial (**C**) orientation on the multiplanar Dyna-CT reconstructions of the *ex-vivo* lamb spine-model. Additionally three-dimensional rendering of the Dyna-CT (**D**) visualizing the trajectory path. The distance between skin entry and target area is indicated (**A**, **C**, and **D**).

In our study the lamb spine was placed in prone position on the interventional table. For hygiene reasons the row meat model was wrapped in cellophane. Before data acquisition a test run was performed to ensure free rotation of the C-arm around the table and safe positioning of the lamb spine. A full series of CT-like slice images with mulitplanar reconstructions were obtained to allow puncture planning and performance with the syngo iGuide® laser guiding system. At the computer workstation the investigator then marked skin entry and puncture target point in the reconstructed slice images of the spine (axial, coronal, and sagittal view) (Figure 1a–c). The first mark being the target area and the second the desired point for needle insertion. Thereby interfering soft tissue structures such as vessels, vital organs or glands could easily be visualized and a different entryway could be found if applicable. Upon this, the indicated trajection channel was re-checked in the coronal and sagittal view. The distance between both marks, resembling the depth of needle insertion, was also indicated (Figure 1a, 1c, and 1d). In a further confirmation step, Syngo iGuide® provided three different views of the planned puncture including a bulls eye view and two other lateral views with different angles. After approving the generated access path, the puncture was performed at the intervention table.

### Puncture with Syngo iGuide® Laser Guidance

**Figure 2:**
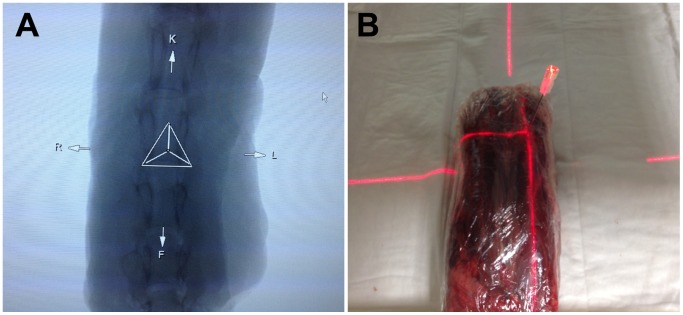
Pre-interventional interventional table positioning and laser-guided puncture. Correct pre-interventional positioning of the free-floating interventional table shown as three lines properly positioned in the indicated triangle (**B**). Correct laser-guided puncture along the planned trajectory indicated by the needle in-line with the 2D laser cross (**B**).

One author (M.F.) performed fifteen independent punctures of different cervical nerve roots ranging from C2/C3 to C6/C7 bilaterally. Therefore we used a BD Quincke Spinal Needle, 20GA, 3.00 IN, 0.9 × 75 mm, (Becton Dickinson GmbH, Heidelberg, Germany). Syngo iGuide® provided the bull’s-eye view on one of the two screens. At first the investigator needed to adjust the free-floating table manually into the right position, shown on one screen as three lines in a triangle (Fig. 2a). The C-arm is equipped with an integrated laser pointer system, which marked the previously planned puncture site with a 2D laser cross on the model. If the needle was inserted keeping this laser cross permanently in-line (Fig. 2b), the needle tip automatically meet the previously planned target structure. By remembering the provided measurement between skin entry site and target point, the investigator could easily estimate the depths of needle insertion.

When believed to be at the target area, the investigator obtained fluoroscopic images in three different angles (bull’s eye view, oblique view, and lateral view) to check for correct position of the needle tip. After confirming appropriate needle tip position, the investigator injected 1 ml of regular CT contrast dye (Imeron 300, Bracco Imaging, Konstanz, Germany) and obtained an additional series of fluoroscopic control images.

### Procedural Analysis

Each nerve root puncture was planned separately. Planning and procedural time were recorded manually with a stopwatch. Tract length, fluoroscopy time, and dose area product (DAP) were acquired from the exam protocol. DAP was measured for the entire procedure (including planning scan) and separately for fluoroscopic controls only. Planning time was defined as time needed from placing the first mark at the workstation until adjustment of the interventional table. Puncture time was defined as time from first needle skin contact until successful placement at the nerve root.

Correct needle placement was analyzed visually by two independent investigators in the three fluoroscopic control images obtained in different angels. To further verify the supposedly correct needle tip position of the fluoroscopic control images, for study reasons, additional MPR Dyna-CT images were acquired. Correct placement was defined as location of the needle tip in the nerve root foramen.

After contrast dye injection correct contrast dye distribution was rated as contrast spread in the ipsilateral nerve root foramen and into the peri-radicular space by evaluation of three fluoroscopic control images. Additively, to validate correct contrast distribution post-injection MPR Dyna-CT images were performed and analyzed, as part of the study.

## Results

**Figure 3:**
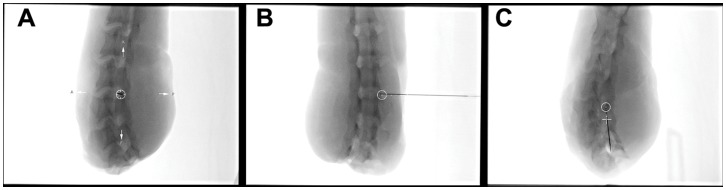
Fluoroscopic control images for correct needle positioning. Bulls’eye view (**A**) and angulated lateral (**B**) and oblique (**C**) views confirming the correct trajectory in-line with the indicated road-map. The positioning of the needle tip centered in the bulls’ eye (**A**, **B**, and **C**) confirming the needle tip in the target area.

**Figure 4:**
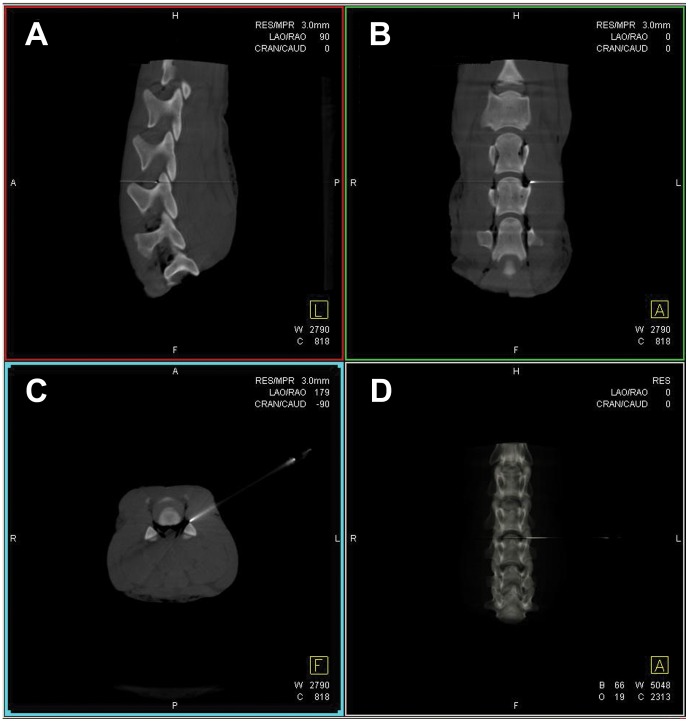
Needle tip localization in post-puncture control Dyna-CT images. Lateral (**A**), coronal (**B**), and axial views (**C**) of the multiplanar reconstructions, as well as (**D**) three-dimensional rendering of the Dyna-CT of the *ex-vivo* lamb cervical spine-model demonstrating the location of the needle tip and the trajectory used to access the neural foramen. The needle tip is located in the posterior part of the neural foramen, anatomically well away from the vertebral vessels but directly adjacent to the path of the nerve root.

**Figure 5:**
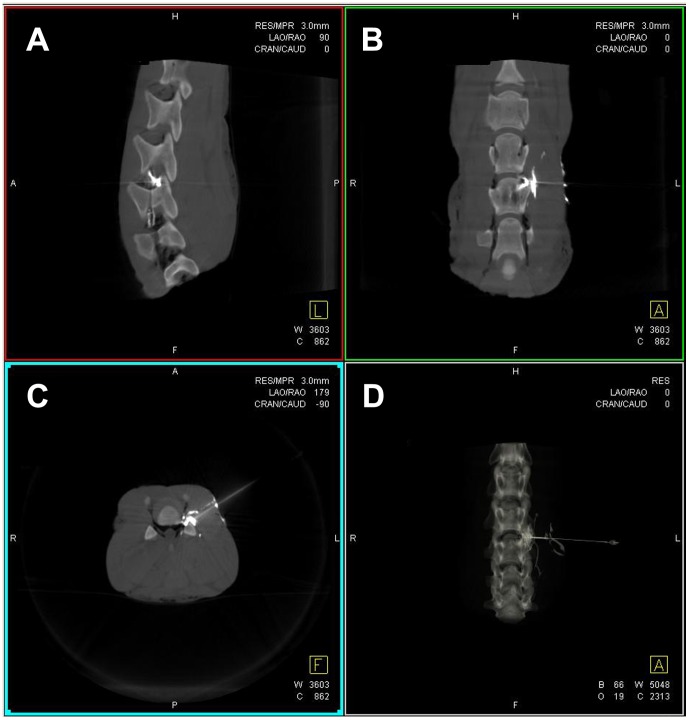
Contrast dye distribution in post-injection control Dyna-CT images. Lateral (**A**), coronal (**B**), and axial views (**C**) of the multiplanar reconstructions, as well as (**D**) three-dimensional rendering of the Dyna-CT of the *ex-vivo* lamb spine-model demonstrating correct distribution of the contrast material within the neural foramen after contrast dye injection.

All 15 punctures were rated as successful by two independent investigators evaluating three fluoroscopic control images (Figure 3a–c) and post-interventional Dyna-CT images (Figure 4a–d) for correct needle positioning and after contrast application for intra- and extraforaminal contrast dye distribution around the cervical nerve root (Figure 5a–d). A second attempt was needed in three punctures to slightly correct the needle position. Punctures 2, 4, and 6–15 were successful on the first attempt.

The required time for Dyna-CT including 3D reconstruction was approximately 1 minute. Mean planning time for the puncture was 2.03 (±0.39) min. mean puncture time was 2.16 (±0.32) min, while mean fluoroscopy time was 0.17 (±0.06) min. Mean tract length was 2.68 (±0.23) cm. Mean total DAP was 397.45 (±15.63) µGy m^2^, while mean total DAP for control fluoroscopy separately was 6.35 (±2.28) µGy m^2^. Detailed data overview is given in Table 1.

**Table 1:** Detailed data for cervical selective nerve root block using Dyna-CT with syngo iGuide® laser-guidance.

	Nerve Root Level	Attempts	Planning time (min:sec)	Puncture time (min:sec)	Tract length (cm)	Fluoroscopy time (min:sec)	Total DAP^1^ (µGy m^2^)	Fluoroscopy DAP (µGy m^2^)
Puncture 1	C3	2	03∶31	03∶26	2.36	00∶29	430.3	10.4
Puncture 2	C4	1	02∶59	03∶02	2.32	00∶20	406.3	7.4
Puncture 3	C5	2	02∶48	03∶14	2.62	00∶26	409.8	9.9
Puncture 4	C6	1	02∶02	02∶24	2.69	00∶19	402.6	7.2
Puncture 5	C4	2	02∶51	02∶14	2.31	00∶25	410.4	10.2
Puncture 6	C7	1	01∶54	02∶24	2.96	00∶20	412.3	7.1
Puncture 7	C5	1	01∶41	01∶54	2.74	00∶13	378.6	4.9
Puncture 8	C6	1	01∶35	02∶01	2.98	00∶12	397.2	5.1
Puncture 9	C3	1	01∶42	01∶53	2.64	00∶14	397.4	4.8
Puncture 10	C4	1	01∶37	02∶07	2.67	00∶12	399.6	5.3
Puncture 11	C5	1	01∶46	01∶56	2.58	00∶11	381.7	4.3
Puncture 12	C7	1	01∶33	01∶52	3.02	00∶11	394.5	4.9
Puncture 13	C3	1	01∶35	01∶55	2.93	00∶12	381.5	4.8
Puncture 14	C4	1	01∶37	01∶46	2.64	00∶13	369.7	4.2
Puncture 15	C3	1	01∶39	01∶54	2.67	00∶12	389.9	4.7

1 DAP: dose area product.

## Discussion

Cervical nerve root blocks have been performed since the late 19^th^ century [27], with a considerable increase in the last decades [28]. This well-established conservative procedure is used to treat and manage resistant cervical radiculopathy, and complex regional pain syndromes. Injections, such as CSNRB attempt to bathe the affected nerve root in steroids and/or local anesthetic [3].

For a long time CSNRB were performed without imaging guidance using palpable anatomic landmarks to direct needle placement. Nowadays, a variety of radiographic techniques are used in clinical practice to minimize complications occurring with blind CSNRB.

CSNRB was conducted in the last decades, also due to a lack of alternatives, using fluoroscopy. However, fluoroscopy is limited in terms of visualizing the soft-tissue structures of the neck. Various complications associated with the fluoroscopic-guided CSNRB including cerebellar infarct, spinal cord infarct, quadriplegia and death [29], [30], [31] have been reported. However, complications associated with cervical injections, such as spinal cord or brainstem damage, seem to be rare with fluoroscopy. One study of a series of more than 1,000 blocks showed a major adverse events rate of less than 1 percent and minor complication rate of 1.66 percent [32]. Nevertheless, the difficulty of this procedure is exacerbated by the fact that fatal complications, especially vascular injuries like dissections, may not cause immediate clinical symptoms and are difficult to visualize with fluoroscopic [33]. In contrast, Nakagawa et al. reported that ultrasound-guidance might be useful for CSNRB by improving nerve and vascular localization [34]. Jee et al. have demonstrated that ultrasound-guided selective nerve root block and fluoroscopy-guided transforaminal block for the treatment of radicular pain in the lower cervical spine are equally effective in pain relief and functional improvement. They found ultrasound provided the benefit of real-time imaging without exposure to radiation [15]. Nevertheless, ultrasound-guidance enables only limited pre-puncture planning and provides inherent technical limitations like the visualization of anatomical structures underneath the bony suface. Pedicelli et al. describe the multi-slice CT-guided approach as the ‘clinical gold standart’ technique, as it provides highly detailed axial images, characterized by high spatial and contrast resolution, but without real time procedure control [20]. They also published that needle trajectory in hypertrophic bony overgrowth was superior using a 3D-rotational angiographic unit compared to fluoroscopy. A recent study performed by Kranz et al. evaluated CT fluoroscopy for cervical interlaminar epidural steroid injection. In 53 injections reviewed, there were no immediate symptomatic complications. A single case of thecal sac penetration was identified, which had occurred during an injection at the C5–C6 level. [35].

In conclusion, it seems to be common sense that the better the pre-puncture planning, the tissue visualization of the target area, and the needle control is, the safer and more reliable the procedure can be done. All prior described techniques for pre-interventional planning, needle control and visualization of the target area have been shown to have some benefits and some drawbacks as described above. Despite attractive features of CT-guided techniques compared to fluoroscopy, including direct visualization of soft-tissue planes, vital organs, neural and vascular structures and the precise anatomic localization with millimeter accuracy, all techniques lack the possibility of multi-dimensional puncture guidance. From the reports described above it seems, greater accuracy of needle visualization and better tissue resolution have to be traded with the inability to have excellent needle control or vise versa.

In contrast, the Dyna-CT has a very short examination time minimizing breathing and movement artifacts. It offers high-resolution images with 3D reconstruction of the target structure. Syngo iGuide® allowed detailed pre-puncture planning and enabled 2D laser-guidance of the puncture. As the Dyna-CT incorporates a fully rotatable angiographic unit, with fluoroscopy, using the indicated road map, it also facilitated an excellent needle control and the possibility of immediate needle correction. This might be very helpful especially in cases of more complicated anatomy, where patients already have had surgery or need multiple injections. Syngo iGuide® allowed detailed pre-puncture planning and enabled 2D laser-guidance of the puncture. As the Dyna-CT incorporates a fully rotatable angiographic unit, with fluoroscopy, using the indicated road map, it also facilitated an excellent needle control and the possibility of immediate needle correction. Miller et al. reported that in their study of CT-guided CSNRB the needle needed to be repositioned more than 3 times in about 34% of their punctures [36]. In our trial of 15 punctures only three needed a second attempt. All 12 other punctures reached the target area on the first attempt. This resulted in a procedure time of approximately 5 minutes, which seems very fast. In literature there is a wide range of procedure times reported for CSNRB, ranging from 6 to 19 minutes for CT fluoroscopy-guided techniques [35], [37]. Nevertheless, it is very difficult to compare procedure times with this trial, as it surely was performed in an *ex-vivo* lamb model. Therefore several time consuming steps did not apply, such as the skin did not needed to be prepped and draped, no anatomical traction through breathing or (un-) intentional movements of the patient had to be regarded and last but not least, there was simply no interaction with a human being.

Undoubtedly, a limitation of 3D puncture planning with laser-guidance compared to ultrasound-guidance is the required use of radiation. The mean total DAP of the cervical nerve root block procedure was calculated with 397.45 µGy m^2^, while the mean total DAP for control fluoroscopy separately was 6.35 (±2.28) µGy m^2^. These values seem to be appropriate in comparison to other interventional studies, e.g. the needle placement in percutaneous vertebroplasty performed by cone-beam computed tomography guidance or conventional fluoroscopy. The study performed by Braak et al. provided DAP values for the CT-guidance of 514 and the fluoroscopy of 174 µGy m^2^ [38]. However, with accurate 3D pre-puncture planning and the laser-guidance system, correction of the needle tip was rarely necessary in this study. Still the restriction of radiation dose has to be kept in mind and acquisition of multiplanar reconstructions during the intervention should be limited to complex procedures or unsuccessful punctures with the standard procedure under fuoroscopic guidance.

Prior evaluations of other laser-guidance systems for CT interventions reported that the usefulness of the guidance device was dependent on the level of experience of the individual physician [39], [40]. Hereby, especially beginners profited by laser guidance systems for standard procedures while experienced interventionalists gained technical help in complex cases. These results are congruent to our experience. The investigator conducting the actual procedure was inexperienced with the technique but felt much more comfortable after the first couple of punctures, which is reflected in a decreasing procedure time a decrease in puncture attempts.

A limitation of this study is the use of only one specimen for puncture feasibility of the laser-guided cervical selective nerve root block with the Dyna-CT. Several specimens with various sizes would have simulated different anatomical conditions.

Furthermore, this study could not determine actual clinical symptom relief. Nevertheless, the puncture approach imitated the technique, which could be used in the clinical setting, with regard of anatomical landmarks and interfering blood vessels. The Artis Zee® Ceiling is completely rotatable around the free-floating full-carbon interventional table. Thus easy access to the puncture site is granted and sterility in clinical practice can easily be maintained.

Therefore the authors of this study determine CSNRB performed with **syngo** iGuide® for Dyna-CT a feasible technique in an *ex-vivo* lamb model.

If syngo iGuide® for Dyna-CT would facilitate to puncture the target nerve root at the first attempt in *in-vivo* models as well, needs to be determined in further studies. If this would be possible in a clinical setting, it could significantly improve clinical practice, reduce cost, and increase patient satisfaction and compliance with CSNRB.

### Conclusion

CSNRB performed with Dyna-CT and iGuide® is a feasible technique in an *ex-vivo* lamb model. Pre-puncture planning with iGuide® is easy and fast and the laser-guidance system ensured very accurate and intuitive puncture control.
